# Pathogenic Potential of Respirable Spodumene Cleavage Fragments following Application of Regulatory Counting Criteria for Asbestiform Fibres

**DOI:** 10.3390/ijerph192416649

**Published:** 2022-12-11

**Authors:** Melinda Gardner, Martyn Cross, Sue Reed, Maggie Davidson, Rick Hughes, Jacques Oosthuizen

**Affiliations:** 1 School of Medical and Health Sciences, Edith Cowan University, 270 Joondalup Drive, Joondalup, WA 6027, Australia; 2 School of Science, Western Sydney University, Bourke Street, Richmond, NSW 2753, Australia; 3 Microanalysis Australia, 5 Alvan Street, Mt Lawley, WA 6050, Australia

**Keywords:** spodumene, cleavage fragments, lithium, aspect ratio, asbestos, occupational health

## Abstract

Health risks from exposure to lithium-bearing spodumene cleavage fragments are unknown. While asbestiform fibres can lead to fibrosis, mesothelioma and lung cancer, controversy remains whether non-asbestiform cleavage fragments, having equivalent dimensions, elicit similar pathologic responses. The mineralogy of respirable particles from two alpha (α)-spodumene concentrate grades (chemical and technical) were characterised using semi-quantitative X-ray diffraction (XRD). Particles were measured using scanning electron microscopy (SEM) and the dimensions (length [L], diameter [D], aspect ratio [AR]) applied to regulatory counting criteria for asbestiform fibres. Application of the current World Health Organization (WHO) and National Occupational Health and Safety Commission (NOHSC) counting criteria, L ˃ 5 µm, D ˂ 3 µm, AR ˃ 3:1, to 10 SEM images of each grade identified 47 countable particles in the chemical and 37 in the technical concentrate test samples. Of these particles, 17 and 16 in the chemical and technical test samples, respectively, satisfied the more rigorous, previously used *Mines Safety and Inspection Regulations 1995* (Western Australia [WA]) criteria, L ˃ 5 µm and D ≤ 1 µm. The majority of the countable particles were consistent with α-spodumene cleavage fragments. These results suggest elongated α-spodumene particles may pose a health risk. It is recommended the precautionary principle be applied to respirable α-spodumene particles and the identification and control of dust hazards in spodumene extraction, handling and processing industries be implemented.

## 1. Introduction

Lithium, the lightest metal, is vital in the modern world due to its unique physical and chemical properties and high electrochemical reactivity [1]. With diverse applications lithium is widely used in glass, ceramics, lubricating greases, industrial air-conditioning, catalysts [2], pharmaceutical therapies [3], and atomic energy and nuclear technologies [4]. In the last ten (10) years demand for lithium has experienced significant growth [5]. This growth has been driven by the development and mass commercialisation of lithium-ion battery technologies to power consumer electronic devices (e.g., computers, mobile phones, tablets) and electric vehicles (EVs) [6,7]. With the rapid adoption of electric vehicles (EVs), almost exclusively powered by lithium-ion batteries, lithium, as lithium carbonate equivalent (LCE), was forecasted to increase by more than 300% between 2017 and 2025 [8].

While most lithium resources are available in brine deposits, lithium-bearing minerals are another major source [9]. Spodumene (LiAlSi_2_O_6_) occurs in coarse-grained pegmatite deposits and co-exists with quartz, feldspar, and micas [10]. Despite the occurrence of lithium in other minerals of economic importance, including petalite, lepidolite and amblygonite [11], spodumene is the most commercially exploited due to its wide distribution, high abundance, and high lithium content (approximately 8.0% lithium oxide [Li_2_O]) [12,13]. Spodumene accounts for ˃ 90% of global production from all mineral-based sources [14] with Australia amongst the world’s largest producers [15].

Spodumene is a complex single-chain aluminium silicate and a member of the pyroxene group [1]. In nature spodumene occurs in alpha (α) form, referred to as α-spodumene, and has a densely packed crystal structure resistant to direct leaching [9]. For effective lithium extraction early stage processing requires α-spodumene to undergo extensive crushing, grinding and milling, known as comminution [16]. Primary, secondary, and tertiary comminution crushing circuits reduce the ore from a maximum block size of up to 1 metre (m) to a gravel of ˂8 millimetres (mm) [16]. However, extensive comminution may expose workers to potentially toxic mineral dust.

Mechanical pressure applied to α-spodumene throughout comminution causes the crystal to fracture along the preferential side {110} and basal {001} planes of weakest bond strength [7,17,18]. As a result, breakage fragments the mass crystal into smaller prismatic particles, known as cleavage fragments [19].

Cleavage fragments differ from asbestos fibres. Asbestos is the commercial term applied to the six regulated fibrous asbestiform minerals of chrysotile, crocidolite, amosite, anthophyllite asbestos, tremolite asbestos, actinolite asbestos [20]. Asbestiform fibres have a unidirectional growth habit and are comprised of bundles of parallel fibres [21]. Individual fibres are made up of even smaller separable crystal fibres known as fibrils [21]. Furthermore, asbestiform fibres display high tensile strength and flexibility often retaining their aspect ratio when split apart [22] with bundles of fibres commonly observed with splayed ends [23]. Moderate grinding of asbestiform fibres does not create cleavage fragments, but rather the separation of fibres and fibrils occurs [22].

In contrast, cleavage fragments have a multidirectional crystalline growth pattern [23] and are weak, brittle and inflexible [20]. Cleavage fragments are not composed of fibres or fibrils [20] but are simply smaller pieces of the mass body of the mineral crystal [22]. However, individual cleavage fragments may exhibit an elongated, splinter-like [21], columnar or acicular (needle-like) morphology [23] microscopically similar to asbestiform fibres [20].

The physical dimensions, length (L), diameter (D) and aspect ratio (AR) (AR: L/D) of mineral fibres is a critical factor in pulmonary toxicity, pathogenicity, carcinogenicity, and inflammation [24,25,26,27]. Long, thin, respirable asbestiform fibres that cannot be efficiently removed or successfully phagocytised by alveolar macrophages (AM) results in macrophage and epithelial injury, persistent inflammation or can migrate to the interstitium or pleura, all ultimately causing disease [24,28,29].

Despite more recent research recognising other determinants including size and shape, chemical composition, surface chemistry and surface charge, and durability in the lung (biopersistence) all influencing the toxicity of mineral fibres [28,30], geometry and dimension are still considered key factors of toxicity [28]. Some studies suggest that irrespective of crystalline structure, dimension and durability are the most influential factors of fibre pathogenicity once deposited in the respiratory tract [31].

Indeed, the concentration of fibres that fulfil specific dimensional metrics is used as the basis for regulatory threshold levels for asbestiform fibre exposure [32]. For regulatory purposes, the World Health Organization (WHO) (1997) defines biologically critical fibres as those ˃5 µm in length, ˂3 µm in diameter with an AR ˃ 3:1 [33]. These same physical parameters are also used as Australian criteria by the National Occupational Health and Safety Commission (NOHSC) (2005) [34].

Many authors propose more rigorous dimensional counting criteria be implemented to better discriminate asbestiform fibres from potentially non-toxic cleavage fragments [35,36,37]. Various metrics have been suggested including particles with a L > 10 μm, D < 0.4 μm, AR > 25:1 [35], D < 1.5 μm, AR > 20:1 [36] or AR ≥ 5:1 [37,38] only be considered as countable. Western Australia (WA) previously used the more stringent dimensional parameters of L ˃ 5 µm and D ≤ 1 µm as counting criteria in the *Mines Safety and Inspection Regulations 1995* (WA) (s.9.33.3) [39]. However, in March 2022 these dimensional parameters were repealed in favour of the more conservative WHO (1997) and NOHSC (2005) criteria with the enactment of the *Work Health and Safety (Mines) Regulations 2022* (WA) [40]. Nevertheless, the more refined metrics specified in the *Mines Safety and Inspection Regulations 1995* (WA) remain beneficial for the proper assessment of any health risks associated with exposure to respirable mineral particles [20].

While the causal relationship between inhalation exposure to asbestiform fibres and adverse health outcomes is well documented, controversy still remains as to whether non-asbestiform elongated mineral particles (EMP) or cleavage fragments, having equivalent dimensions, elicit a similar pathologic response [19]. There is a large body of evidence indicating that cleavage fragments and non-asbestiform EMP do not pose the same health risk as asbestiform fibres [20,41,42,43]. However, some studies suggest that inhaled cleavage fragments from non-fibrous minerals may be potentially toxic [44,45].

With the rapid growth in global demand for lithium and lithium-rich compounds over the last decade lithium recovery from spodumene has garnered much interest from industry [46]. Several studies have reported on the optimisation of spodumene lithium recovery with a focus on phase transition [1,47], grinding methods [6,18], floatation conditions [2,4,10,13,14,17] decrepitation techniques [47], novel reagent development [48], and surface chemistry [2,17,49], however, the unique hazards workers may be exposed to in spodumene mining, handling and processing industries are largely ignored.

There is no data available in published literature on the potential toxicity or pathogenicity of α-spodumene particles in vitro or in exposed humans or experimental animals. In addition, α-spodumene is insoluble in water and dilute acids [50]. Dissolution is documented to only occur in strong acids [51,52] suggesting that incomplete clearance may result in persistence in the lung following exposure. As the demand for lithium experiences significant growth, so too are potential adverse health effects arising from occupational inhalation exposure to α-spodumene dust during mining or production processes.

This study aims to determine the dimensional characteristics (length, diameter or width, and aspect ratio) of cleavage fragments generated from typical comminution (crushing/grinding) processes and apply regulatory counting criteria in accordance with WHO (1997) and NOHSC (2005) [33,34], L ˃ 5 µm, D ˂ 3 µm, AR ˃ 3:1, and the more stringent, L ˃ 5 µm and D ≤ 1 µm, previously used as counting criteria by the *Mines Safety and Inspection Regulations 1995* (WA) [39]. Geometry and dimension are shown to be key factors in the carcinogenic [25,27], pathogenic, cytotoxic [30] and inflammatory effects [53,54] of mineral fibres.

## 2. Materials and Methods

### 2.1. Materials

Two types of test materials, chemical and technical grade α-spodumene concentrate, were collected from a spodumene mining and processing facility in WA, Australia. The α-spodumene ore was recovered from a single pegmatite deposit.

Approximately 1.5 kilograms (kg) of each concentrate grade was collected directly from their respective processing conveyors following comminution and beneficiation, to remove major impurities, also known as gangue materials. Both grades of crushed α-spodumene concentrate test samples were observed as white to grey in colour and resembled damp, fine sand. The concentrates had a high water content due to the wet comminution and heavy liquid separation and floatation techniques used for beneficiation.

The two test samples were selected as being representative of relatively high-grade α-spodumene concentrate accompanied by typical gangue materials. The two sample grades were selected for the variance in Li_2_O content found across and within spodumene deposits which may influence cleavage fragment morphology.

In general, chemical grade α-spodumene concentrate has an average Li_2_O content of approximately 6.0% and a maximum ferric oxide (Fe_2_O_3_) content of 1.0%. Chemical grade concentrate is mainly used in the manufacture of lithium-ion batteries, lubricants, refrigeration, metal alloys, optical glass, pharmaceuticals and polymers. In contrast, technical grade α-spodumene concentrate typically has a higher purity of Li_2_O (˃7.0%), significantly lower Fe_2_O_3_ content and a lesser number of gangue materials. Technical grade concentrate finds use as an additive in tableware, container and specialty glass products. Both the chemical and technical grade α-spodumene concentrate test samples were treated separately.

### 2.2. Methods

#### 2.2.1. Mineral Characterisation

To analyse the composition and identify the concentration of α-spodumene in the test materials a 2.7 grams (g) sub-sample was removed from each grade and micronised in a McCrone Micronising Mill. Sub-samples were subjected to light grinding such that 90% were sieved to pass ≤ 20 µm and dried at 40 °C overnight. The mineralogy was determined using semi-quantitative X-ray diffraction (XRD) analysis with a corundum spike. XRD data were processed using Eva 4.3 search match software. Crystalline mineral compounds were calculated using the normalised reference intensity ratio (RIR) method at a wavelength (λ) of 1.78901 Angstrom (Å). It should be noted that XRD scan only gives peaks for crystalline materials while amorphous material adds to the background.

#### 2.2.2. Measurement of Cleavage Fragment Sizes Use a Scanning Electron Microscope (SEM)

In preparation for undertaking SEM analysis, 10 g of each spodumene grade were passed through horizontal elutriation to extract respirable particles with an EAD ≤ 4 µm. A small portion of the elutriated particles was removed from each grade and mounted on top of separate double-sided carbon tabs. Samples were not carbon coated. Carl Zeiss EVO50 scanning electron microscope (SEM) fitted with Oxford INCA X-Max energy dispersive spectrometer (EDS) was used to identify EMP. SEM operating conditions were 20 kilo electron volts (KeV) working at a magnification of ×2000. Backscattered electron images (BEI) were captured, and each image frame individually examined. Every countable particle in the images was manually measured using the digital calipers in the Oxford INCA software to determine length and diameter. The AR of the countable particles was then calculated.

Following NOHSC:3003 (2005) *Guidance Note on the Membrane Filter Method for Estimating Airborne Asbestos Fibres*, particles with dimensions that satisfy counting criteria by WHO (1997) and NOHSC (2005), L ˃ 5 µm, D ˂ 3 µm, AR ˃ 3:1, and the previously used counting criteria stipulated by *Mines Safety and Inspection Regulations 1995* (WA), L ˃ 5 µm and D ≤ 1 µm, were determined.

## 3. Results

### 3.1. Mineral Characterisation

**Figure 1:**
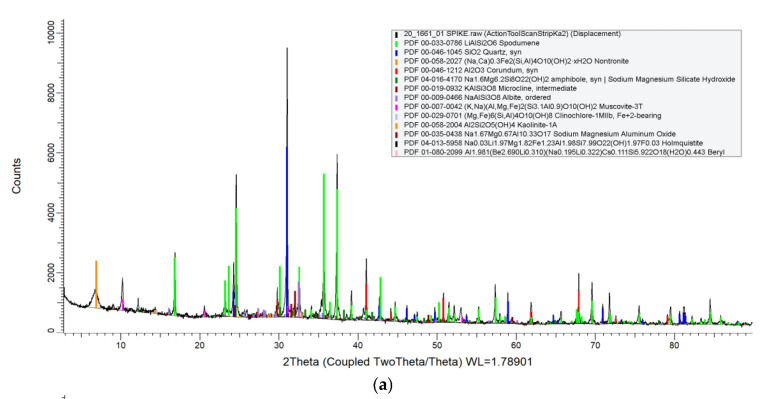

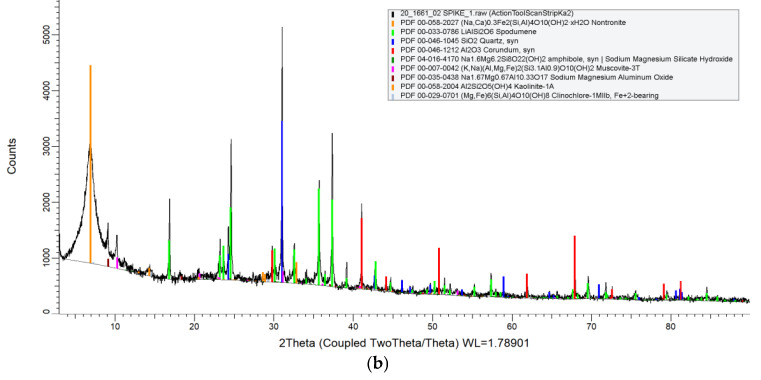
(**a**) XRD spectra of minerology of crystalline particles in chemical grade α-spodumene concentrate test sample, (**b**) XRD spectra of minerology of crystalline particles in technical grade α-spodumene concentrate test sample.

The mineralogy of crystalline particles identified in the test materials by XRD results are summarised in Table 1. The mineralogy is listed in order of concentration as a relative percentage (weight %) of each phase based on the contribution to the sum. Corresponding XRD spectra of the mineral compounds are shown in Figure 1. International Centre for Diffraction Data (ICDD) match probability (i.e., major, good, medium, minor, low, trace) for each compound is reported as indicative based on similar peak positions and relative intensities following comparison to data in published literature.

Due to low concentrations full characterisation of the amphibole and sodium magnesium aluminium oxide present in both concentrate grades could not be determined with certainty. Generic amphibole and sodium magnesium aluminium oxide XRD patterns were selected to complete the minerology.

Spodumene was confirmed as the most abundant mineral in the chemical grade concentrate accounting for 42%, followed by quartz at a significantly lower concentration at 14%. Both spodumene and quartz observed good ICDD match probability. The remainder of the chemical grade concentrate sample was completed by nontronite, feldspars (albite and microcline), mica (muscovite), clay minerals (clinochlore and kaolinite) and holmquistite and beryl in medium, low, or trace accessory phases.

In the technical grade sample, nontronite was the most dominate mineral phase at 52%, followed by spodumene and quartz at 25% and 12%, respectively. The remainder of the technical grade concentrate was completed by muscovite, amphibole and clay minerals as minor or trace accessory phases. No asbestiform minerals were identified in either of the concentrate test sample grades.

### 3.2. Identification and Measurement of Countable Particles Using SEM

**Figure 2:**
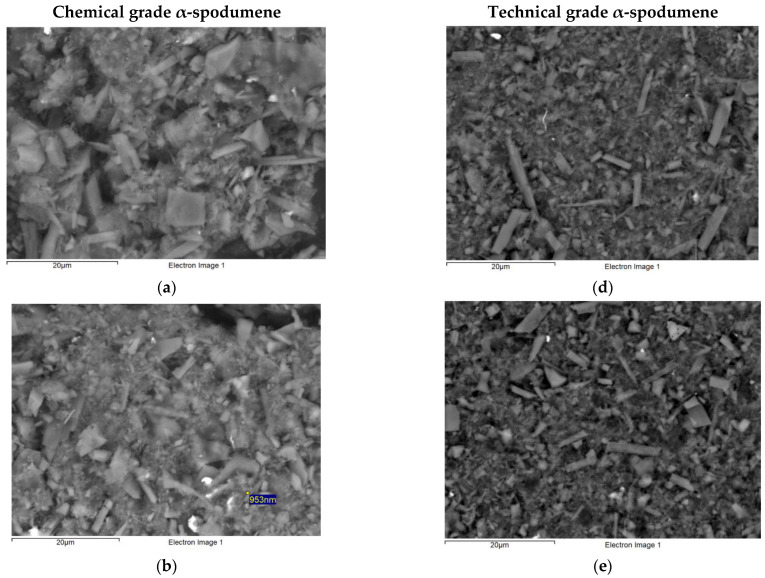

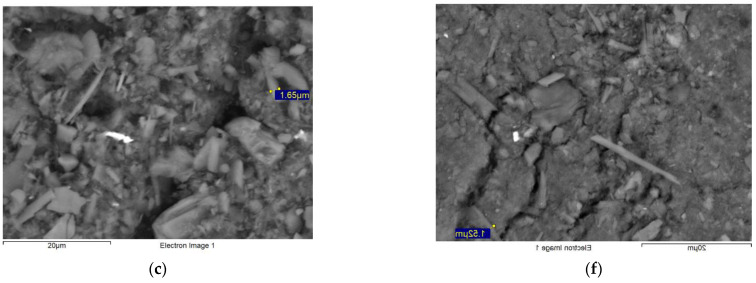
SEM images of α-spodumene concentrate samples following typical comminution and beneficiation for chemical grade (**a**–**c**) and technical grade (**d**–**f**) illustrate the morphology of EMP with the majority exhibiting stepped sides with blunt or stepped ends consistent with α-spodumene cleavage fragments.

A total of 20 images were collected from both grades of prepared concentrate, ten for chemical grade and ten for technical grade. For comparison, Figure 2 shows a representative selection of the SEM images from each of the grades.

In the 10 images examined of chemical grade concentrate, 47 particles were identified as satisfying counting criteria according to WHO (1997) and NOHSC (2005). Of these 47 particles, 17 were countable by the *Mines Safety and Inspection Regulations 1995* (WA) criteria. Using the WHO (1997) and NOHSC (2005) criteria, in the 10 images examined of the technical grade concentrate 37 particles were countable. Of these particles, 16 were countable by the *Mines Safety and Inspection Regulations 1995* (WA) criteria. Table 2 summarises the number of countable particles measured within the 10 images of each sample grade that satisfied the two different counting criteria.

Following the WHO (1997) and NOHSC (2005) counting criteria, measurement data from the SEM images identified countable particles in the chemical grade concentrate sample exhibited a mean (M) L 7.9 µm (M = 7.9) (range 5–20) µm, mean D 1.1 µm (M = 1.1) (range 0.2–2.2) µm and a mean AR 9:1 (M = 9:1) (range 3:1–32:1). Using the same counting criteria, countable particles in the technical grade demonstrated a mean L 7.6 µm (M = 7.6) (range 4.2–18.1) µm, mean D 1.1 µm (M = 1.1) (range 0.1–2.5) µm, mean AR 10:1 (M = 10:1) (range 3:1–65:1).

Following application of the *Mines Safety and Inspection Regulations 1995* (WA) counting criteria, SEM measurements determined that countable particles in the chemical grade sample exhibited a mean L 7.5 µm (M = 7.5) (range 5–12.7) µm, mean D 0.6 µm (M = 0.6) (range 0.2–1) µm, and mean AR 14:1 (M = 14:1) (range 7:1–32:1) µm. The same counting criteria applied to the technical grade sample demonstrated a mean L 7.1 µm (M = 7.1) (range 5–18) µm, mean D 0.6 µm (M = 0.6) (range 0.1–1) µm and a mean AR 17:1 (M = 17:1) (range 7:1–65:1). A complete summary of dimensional (µm) statistics for the countable particles is shown in Table 3.

## 4. Discussion

This study has identified that the typical comminution used in α-spodumene processing readily generates cleavage fragments that satisfy the widely used WHO (1997) and NOHSC (2005) regulatory counting criteria and the previously used *Mines Safety and Inspection Regulations 1995* (WA) criteria. Details of the counting criteria outcomes are shown in Table 2 with the summary of measurement data dimensions (µm) for countable particles presented in Table 3.

Despite both chemical and technical grade concentrates having completed beneficiation, XRD study results revealed a complex mineralogy as shown in Table 1. In both concentrate test samples, α-spodumene, quartz and nontronite were the three major phases. In particular, the chemical grade concentrate identified α-spodumene to be the dominant phase at 42%, and the second most dominant phase in the technical grade at 25%. In the technical grade concentrate, nontronite was shown to be the most abundant, making up 52% of the sample. The chemical grade test sample also demonstrated a greater number of gangue materials with the occurrence of microcline, albite, holmquistite and beryl which were below detection limit (BDL) in the technical grade sample.

Due to pronounced cleavage planes, α-spodumene cleavage fragments typically appear long, flat or columnar in shape with stepped, bladed or prismatic surfaces [7,18,55] while very fine cleavage fragments can be observed as long needle-like splinters [55]. In contrast, the mechanical fracture of quartz exposes many singular planes as it lacks distinct cleavage planes [56,57]. This absence of cleavage planes generates particles that are characterised as highly irregular in shape with sharp protruding edges and acute spikes [56,58].

In general, crystalised nontronite forms globular or spheroidal aggregates composed of intergrown fibre-like or lath-shaped particles [59]. Depending on geographical location, particles may reach 10–100 µm in length and 1 µm in width [59,60]. Well-developed ribbon-like nontronite particles often exhibit smaller dimensions and may only reach sizes up to 1 µm in length and 0.25 µm in diameter [60]. Microscopically, nontronite particles are commonly observed with frayed or fuzzy edges likely due to their layered, sheet structure [60,61]. Large separation of the layered stacks is more predominant when particles are viewed in aqueous solution [61], however, the layers tend to collapse with water loss [62]. Dense particles with irregular or prismatic outlines may also appear dispersed within nontronite aggregates often in sub-µm size ranges [60].

As shown in Figure 2, SEM images revealed a wide range of particle morphologies. The bulk of the chemical grade concentrate appeared coarse in grain with the majority of individual particles irregular or block-like in shape, indicative of quartz or α-spodumene cleavage fragments. Of the countable particles, a large portion exhibited a lath, acicular, splinter- or needle-like morphology, stepped sides and blunt or stepped ends, also consistent with α-spodumene cleavage fragments. A small number of countable particles were observed with a fibre-like morphology indicative of holmquistite. Holmquistite is an amphibole commonly occurring as aggregates at the contact between lithium-bearing pegmatites and surrounding rocks [63]. Holmquistite aggregates can sometimes appear as fibrous textured in sheaf-like masses of small acicular crystals [64].

SEM images (Figure 2) of the technical grade concentrate demonstrated an overall finer grain with the bulk of particles more irregular or columnar in shape. However, the countable particles were similar to those observed in the chemical grade with the majority consistent with α-spodumene cleavage fragments. None of the countable particles in either grade concentrate presented with a lamellar, plate or sheet morphology or were observed with frayed or fuzzy edges which may be indicative of nontronite, clay or mica minerals.

Comparison of measurement results revealed countable particles in the two grades exhibited similar size ranges as recorded in Table 3. The similar dimensions observed in both test samples grades indicates that the higher Li_2_O purity and lower Fe_2_O_3_ content in technical grade α-spodumene concentrate does not influence the dimensions of cleavage fragments. However, a single countable particle in the technical grade concentrate sample was measured with a significantly greater maximum AR, more than two-fold, when compared to the maximum AR in the chemical grade sample. An explanation for the difference in maximum AR may be due to the larger concentration of nontronite in the technical grade sample. Nontronite can produce particles with a fibre-like morphology reaching lengths between 10–100 µm and 1 µm in width depending on the deposit [59,60]. The absence of layered stacks or fuzzy edges, often observed with nontronite particles, may be due to water loss from the test sample.

**Figure 3:**
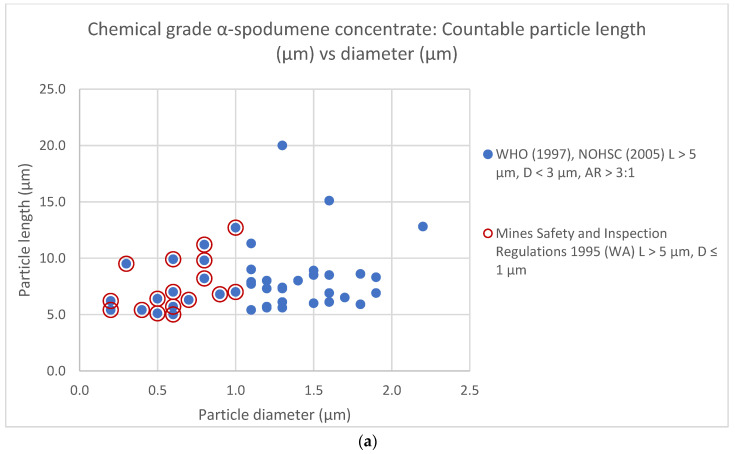

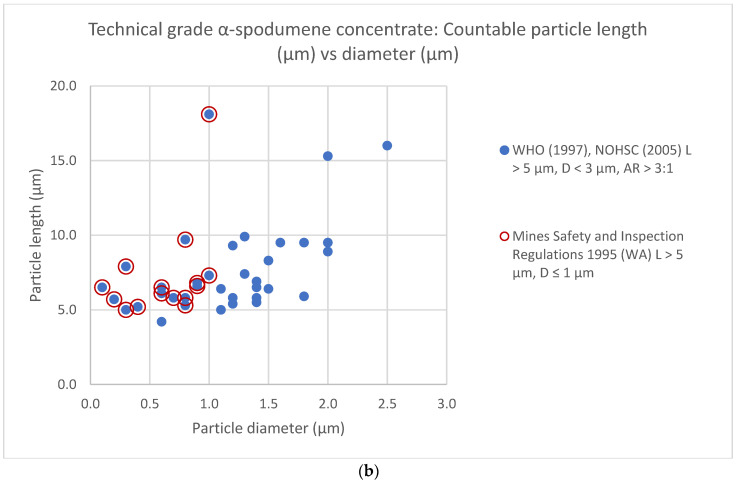
Distribution of length and diameter (µm) data for countable particles in the (**a**) chemical grade α-spodumene concentrate test sample and (**b**) technical grade α-spodumene concentrate test sample.

A scatterplot of the distribution of L and D (µm) measurements of countable particles that satisfied the two different counting criteria in both α-spodumene concentrate grade test samples is shown in Figure 3.

For several decades extensive research has been dedicated to understanding the mechanisms of disease induction following the inhalation of naturally occurring mineral fibres [65,66,67,68,69,70,71,72]. There is strong evidence indicating that length is the critical factor in determining the carcinogenic potential of mineral fibres [30,53,54,73] rather than mineralogical origin or chemical composition [19,54].

As proposed in the widely used “Stanton Hypothesis”, needle-shaped particles with a L > 8 µm and D ≤ 0.25 µm are the optimal dimensions for the induction of intrapleural tumours [26]. Based on experimental animals and using a variety of particles, including both fibrous and non-fibrous elongated particles, Stanton et al. (1981) further observed a relatively high correlation with probability of pleural sarcoma from long, thin particles with a L ˃ 4 µm and D ˂ 1.5 µm. The particles studied seemed to only share dimensional characteristics and durability as common factors [26].

Measurement data identified no countable particles in either the chemical or technical grade concentrate samples satisfied the optimal physical parameters detailed by the “Stanton Hypothesis”. However, application of the WHO (1997) and NOHSC (2005) counting criteria, determined that 70.2% of countable particles in the chemical grade sample and 75.7% of countable particles in the technical grade samples met with the dimensional parameters, L ˃ 4 µm and D ˂ 1.5 µm, as reported by Stanton et al. (1981) as correlating with a relatively high probability of carcinogenicity.

Lippman (1990) and Donaldson et al. (1992) support that fibre length is a critical determinant of pathogenicity [53,74]. According to Lippman (1990), a critical length range > 10 µm is necessary for pathogenicity and lung fibrogenesis for fibrous minerals [74]. Following intratracheal instillation in experimental animals Lippman (1990) states that lung fibrosis is produced from virtually all fibrous minerals with L > 10 µm [74]. In addition, Donaldson et al. (1992) observed that long asbestiform amosite fibres, with a 40% population L ˃ 10 µm, demonstrated greater injury and inflammation activity in male mice following intraperitoneal injection when compared to short amosite fibres with similar diameters [53].

To expand on the importance of length in fibre pathogenicity, recent research by Padmore et al. (2017) reported that length is a critical factor in fibre-induced cytotoxicity as well as increased inflammatory response in AM [75]. In the study, murine AM were exposed in vitro to short and long populations of entangled uncoated borosilicate glass fibres (JM-100 glass fibres) with nominal diameters between 0.1 and 10 μm [75]. Glass fibres were selected to isolate the single physical parameter of length and to minimise the influence of surface chemistry on fibre-cell interaction [75]. Padmore et al. (2017) concluded that although short glass fibres with mean L = 7.0 μm were more readily internalised by macrophages when compared to long fibres L = 39.3 μm, short fibres played a minor role in inflammatory biomolecule production in exposed AM [75].

Despite α-spodumene not occurring in fibrous habit, the measurement data shown in Table 3 established that a total of 12.8% countable particles in the chemical grade sample and 8.1% of particles in the technical grade sample exhibited a L ˃ 10 µm as reported by Lippman (1990) and Donaldson et al. (1992) as a determinant of carcinogenic and fibrogenic potency. Furthermore, overall countable particles in the chemical and technical grade samples demonstrated mean L = 7.9 µm and L = 7.6 µm, respectively. As described by Padmore et al. (2017), this suggests that while respirable α-spodumene cleavage fragments in this size range may be more readily phagocytised, based on length alone they may also increase inflammatory biomolecule production in AM [75].

Further studies have investigated fibre length as a determinant of toxicity using *in vivo* and/or in vitro mouse and rat bioassays following exposure to natural and synthetic fibres including but not limited to JM-100 glass fibres (mean L = 3, 4, 7, 17, 33 µm) [76], short (mean L = 1.2 µm), long (mean L = 5.4 µm) and mixed (mean L = 2.8 µm) crocidolite asbestos fibres [77], glass microfibre, silicon carbide whisker and amosite asbestos (length categories 5–10, 10–15, ˃15 µm) [78], glass microfibre, silicon carbide whisker, amosite asbestos, man-made vitreous fibres (MMVFs) and refractory ceramic fibres (RCFs) (length categories ˃0.4, ˃5, ˃8, ˃10, ˃15, ˃20 µm) [79], and silver nanowires (mean L = 3, 5, 10, 14, 28 µm) [80]. In addition, the role of length parameters have been reported using human macrophage and immortalised mesothelial cell lines exposed to carbon nanotubes (1–2, 1–5, 5–20 µm and mean L = 13, 36 µm) [81] and human alveolar macrophages collected from healthy male subjects following exposure to length-classified JM-100 glass fibres (8, 10, 16,20 μm) [82]. However, a consensus is yet to be reached that quantifies a critical length threshold across fibrous materials [75].

More recent research has attempted to model and predict the carcinogenic potency of elongated amphibole particles based on dimensional characteristics and fibrous versus non-fibrous mineral growth habit [25,27]. Wylie et al. (2020) demonstrated a strong correlation with mesothelioma potency and fibres with L > 5 µm, width ≤ 0.25 µm, AR ≥ 3:1 using amphibole EMP from four mining and milling cohorts [27]. However, predicted mesothelioma potency following exposure to non-asbestiform amphibole EMP or cleavage fragments were found to be low or not significant [27]. Nevertheless, the low or non-significant relationship was based on a significantly low percentage of average widths satisfying the width criterion.

Rather than length, Korchevskiy and Wylie (2021) proposes that width is a better predictor of mesothelioma and lung cancer potency [25]. Using epidemiological data and the detailed size information of asbestiform, non-asbestiform and mixed habit amphibole particles the authors found fibres with L ˃ 5 µm and width ˂ 0.2 µm had the highest correlation with mesothelioma potency while width ˂ 0.28 µm demonstrated highest correlation with lung cancer [25].

Application of the dimensions, L > 5 µm, width ≤ 0.25 µm, AR ≥ 3:1 for predictive cancer potency, as described by Wylie et al. (2020) and the Korchevskiy and Wylie (2021) width criteria of ≤0.25 µm and ˂0.28 µm to the measurement data confirms that 4.3% of overall countable particles in the chemical grade sample and 8.1% in the technical grade sample satisfied these metrics. However, Korchevskiy and Wylie (2021) reported a lower or non-existent cancer potency from the simulated and raw datasets of 5058 non-asbestiform cleavage fragments compared to the asbestiform varieties.

When compared to asbestiform mineral fibres, cleavage fragments are typically shorter and thicker, break along cleavage planes and lack strength and flexibility [42]. However, a major point of discussion exists as to whether cleavage fragments that meet specific dimensional criteria pose the same health risk [83]. There is strong evidence indicating that mineral cleavage fragments are less pathogenic than asbestiform mineral fibres [19,20,41,42,43]. Roggli (2018) contends that non-asbestiform minerals are not associated with asbestos-induced diseases and, thus, remain unregulated by the Occupational Safety and Health Administration (OSHA) [31].

In contrast, evidence indicating a carcinogenic risk from exposure to cleavage fragments is provided by Allen et al. (2014) [84]. In the case/control study of Minnesota taconite workers, Allen et al. (2014) identified significantly higher than expected mortality rates for lung cancer (Standard Morality Rate [SMR] = 1.16) and mesothelioma (SMR = 2.77) in the taconite worker cohort when compared to the general Minnesota population [84]. Similarly, Germine and Puffer (2015) concluded that most of the fibres in lung tissue of Quebec mine workers who died of mesothelioma were a product of cleavage fragments or parting fragments [85].

Case (1991, p. 357) states that ”For regulatory and health assessment purposes… there is no evidence that potentially affected cells can distinguish between ‘asbestiform’ and ‘non-asbestiform’ fibres having equivalent dimensions” [83]. NIOSH (2011) and the US Environmental Protection Agency (EPA) (2003) also conclude that given equivalent dimensions and similar biopersistence there is no convincing evidence cleavage fragments may be less toxic [19,86]. While there may be limited evidence to suggest that α-spodumene cleavage fragments are potentially pathogenic, due to the uncertainty it is recommended that the precautionary principle be applied. The precautionary principle advises that precautionary measures be taken even if cause and effect relationships are not fully established by scientific evaluation [87].

Results from XRD study (Table 1) demonstrated that post-beneficiation α-spodumene concentrate is a complex mixture of crystalline mineral particles. In addition, XRD results confirmed crystalline quartz (silica) occurs in both concentrate test sample grades in significant quantities. A large body of medical and epidemiological research has established exposure to respirable silica results in or contributes to such diseases as silicosis, cardiovascular disease [88], renal disease [89], carcinoma [90] and immune-mediated disease, including rheumatoid arthritis [91], systemic lupus erythematosus [92] and systemic sclerosis [93]. The overwhelming evidence on silica induced pathogenesis prompted the International Agency for Research on Cancer (IARC) to classify crystalline silica as a ‘Group 1’ carcinogen, that there is sufficient evidence of carcinogenicity in humans and experimental animals [94].

Irrespective of the toxic potential of α-spodumene cleavage fragments, at sufficient airborne concentrations the mineral dust generated by the extraction, handling, and processing of α-spodumene may cause permanent, disabling or fatal respiratory disease due to respirable crystalline silica content. In Australia and the US the 8 h time weighed average (TWA) workplace exposure standard (WES) for respirable crystalline silica dust is 0.05 milligrams per cubic metre (mg/m^3^) with an action level at 0.025 mg/m^3^ [95,96].

## 5. Conclusions

This study has identified that typical comminution processes readily generates particles that satisfy the dimensional parameters of two different counting criteria in both chemical and technical grade α-spodumene concentrate test samples. SEM measurement data revealed that the countable particles exhibited dimensions that correlate with increased AM inflammatory biomolecule production and carcinogenic and fibrogenic potency previously associated with fibrous materials. The majority of these countable particles were consistent with α-spodumene cleavage fragments.

There is some evidence to suggest that long, thin, non-asbestiform EMP or cleavage fragments may lead to mesothelioma and lung cancer and increase the probability of pleural sarcoma in humans and experimental animals. Thus, the extraction, handling, and processing of α-spodumene may generate harmful mineral dust exposing workers to increased risk of developing permanent respiratory disease.

Currently, there is no epidemiological or toxicological data available in the literature reporting on the potential health effects arising from exposure to respirable α-spodumene cleavage fragments. Therefore, a conservative approach is recommended, and the precautionary principle be applied until further research concludes respirable α-spodumene cleavage fragments are non-hazardous to human health. As a precautionary measure, occupational hygiene monitoring should be conducted, in accordance with asbestiform fibre standards, and regulatory counting criteria continue to be implemented to ensure the risk of elongated α-spodumene cleavage fragments is reduced and maintained to as low as reasonably practicable (ALARP).

In addition, respirable crystalline quartz occurs in α-spodumene concentrate in significant quantities. Occupational exposure to respirable crystalline silica dust is documented to cause permanent and fatal respiratory disease. It is further recommended that the identification and control of dust hazards in spodumene extraction, handling and processing industries be implemented to ensure the risk of mineral dust exposure is reduced and maintained ALARP. It is proposed that the robust measures required for the control of respirable crystalline silica should also mitigate any adverse health risks resulting from exposure to respirable α-spodumene cleavage fragments.

## Figures and Tables

**Table 1:** Mineralogy, abundance and ICDD match probability of crystalline minerals in chemical and technical grade α-spodumene test samples identified by XRD.

	Chemical Grade		Technical Grade	
Crystalline Mineral Phase	Conc. (%)	ICDD Match	Conc. (%)	ICDD Match
Spodumene (LiAlSi_2_O_6_)	42	Good	25	Major
Quartz, syn (SiO_2_)	14	Good	12	Major
Nontronite ((Na,Ca)_0.3_Fe_2_(Si,Al)_4_O_10_(OH)_2_·xH_2_O)	14	Medium	52	Major
Microcline, intermediate (KAlSi_3_O_8_)	8	Medium	*BDL	
Albite, ordered (NaAlSi_3_O_8_)	5	Medium	*BDL	
Muscovite-3T ((K,Na)(Al,Mg,Fe)_2_(Si_3.1_Al_0.9_)O_10_(OH)_2_)	3	Medium	3	Minor
Amphibole, syn | Sodium Magnesium Silicate Hydroxide (Na_1.6_Mg_6.2_Si_8_O_22_(OH)_2_)	3	Medium	1	Trace
Clinochlore-1MIIb, Fe^+2^-bearing ((Mg,Fe)_6_(Si,Al)_4_O_10_(OH)_8_)	1	Low	1	Minor
Kaolinite-1A (Al_2_Si_2_O_5_(OH)_4_)	1	Low	2	Minor
Sodium Magnesium Aluminium Oxide (Na_1.67_Mg_0.67_Al_10.33_O_17_)	1	Low	2	Minor
Holmquistite (Na_0.03_Li_1.97_Mg_1.82_Fe_1.23_Al_1.98_Si_7.99_O_22_(OH)_1.97_F_0.03_)	Trace	Low	*BDL	
Beryl (Al_1.981_(Be_2.690_Li_0.310_)(Na_0.195_Li_0.322_)Cs_0.111_Si_5.922_O_18_(H_2_O)_0.443_)	Trace	Low	*BDL	
Amorphous	8		2	

*BDL = Below detection limit.

**Table 2:** Counting criteria outcomes.

Counting Methodology	Chemical Grade	Technical Grade
WHO (1997), NOHSC (2005) L ˃ 5 µm, D ˂ 3 µm, AR ˃ 3:1	47	37
*Mines Safety and Inspection Regulations 1995* (WA) L ˃ 5 µm, D ≤ 1 µm	17	16

**Table 3:** Summary of statistics for countable particles in chemical and technical grade α-spodumene concentrate samples that satisfy the two different counting criteria.

	WHO (1997), NOHSC (2005) L > 5 µm, D < 3 µm, AR ˃ 3:1						*Mines Safety and Inspection Regulations 1995* (WA) L ˃ 5 µm, D ≤ 1 µm					
	Chemical Grade			Technical Grade			Chemical Grade			Technical Grade		
	Length (µm)	^1^ Dia. (µm)	^2^ AR (n:1)	Length (µm)	^1^ Dia. (µm)	^2^ AR (n:1)	Length (µm)	^1^ Dia. (µm)	^2^ AR (n:1)	Length (µm)	^1^ Dia. (µm)	^2^ AR (n:1)
Mean	7.9	1.1	9	7.6	1.1	10	7.5	0.6	14	7.1	0.6	17
Median	7.3	1.2	7	6.5	1.1	7	6.8	0.6	12	6.3	0.7	11
Mode	5.4	1.3		5.8	1.4		5.4	0.6		5.8	0.8	
Standard Deviation	2.8	0.5	6.5	3.1	0.6	11.3	2.3	0.3	7.8	3.2	0.3	14.9
Minimum	5.0	0.2	3	4.2	0.1	3	5.0	0.2	7	5.0	0.1	7
Maximum	20.0	2.2	32	18.1	2.5	65	12.7	1.0	32	18.1	1.0	65
Count	47	47	47	37	37	37	17	17	17	16	16	16

^1^Dia. = Diameter; ^2^ AR = Aspect Ratio.

## Data Availability

Not applicable.
